# Comparative morphometric analysis of lungs of the semifossorial giant pouched rat (*Cricetomys gambianus*) and the subterranean Nigerian mole rat (*Cryptomys foxi*)

**DOI:** 10.1038/s41598-020-61873-8

**Published:** 2020-03-23

**Authors:** John N. Maina, Casmir O. Igbokwe

**Affiliations:** 1 Department of Zoology, University of Johannesburg, Auckland Park Campus, Kingsway, Johannesburg, 2006 South Africa; 2 Visiting Postdoctoral Fellow, Department of Veterinary Anatomy, Faculty of Veterinary Medicine, University of Nigeria, Nsukka, Nigeria

**Keywords:** Biophysics, Computational biophysics, Evolutionary ecology

## Abstract

Lungs of the rodent species, the African giant pouched rat (*Cricetomys gambianus*) and the Nigerian mole rat (*Cryptomys foxi*) were investigated. Significant morphometric differences exist between the two species. The volume of the lung per unit body mass was 2.7 times larger; the respiratory surface area 3.4 times greater; the volume of the pulmonary capillary blood 2 times more; the harmonic mean thickness of the blood-gas (tissue) barrier (τht) ~29% thinner and; the total pulmonary morphometric diffusing capacity (DLo_2_) for O_2_ 2.3 times more in *C. foxi*. *C. gambianus* occupies open burrows that are ventilated with air while *C. foxi* lives in closed burrows. The less morphometrically specialized lungs of *C. gambianus* may be attributed to its much larger body mass (~6 times more) and possibly lower metabolic rate and its semifossorial life whereas the ‘superior’ lungs of *C. foxi* may largely be ascribed to the subterranean hypoxic and hypercapnic environment it occupies. Compared to other rodents species that have been investigated hitherto, the τht was mostly smaller in the lungs of the subterranean species and *C. foxi* has the highest mass-specific DLo_2_. The fossorial- and the subterranean rodents have acquired various pulmonary structural specializations that relate to habitats occupied.

## Introduction

About 300 of the extant mammalian species that represent 54 genera and belong to 10 families of four orders live in moist and dark, climatically stable, hypoxic and hypercapnic underground burrows^1–8^. Such animals inform on the natural evolutionary process of adaptation that has permitted underground life^9^. In synapsids, the lineage that includes modern mammals and their ancestors^10,11^, the extinct mammal-like carnivore, the Cynodont (*Thrinaxodon liorhinus*) which inhabited the Karoo of South Africa ~251 million years ago (mya), is apparently the oldest known burrowing animal^12^. Independently and at different amounts of time, animals invaded the underground ecotope, mostly between the upper Eocene (45–35 mya) and the Quaternary (~2 mya), when the global climate changed markedly to a colder and drier Earth^8,9,13–17^. Predator avoidance, escape from extreme environmental conditions above the ground and gaining access to the subterranean parts of plants like roots, tubers, bulbs and corms and soil invertebrates were the main driving pressures for relocating to underground life. Extremophiles are life forms that have adapted to inhabiting exceptionally exacting conditions which are injurious to conventional living things^18–21^. The hypoxic- and hypercapnic conditions in the unventilated, perpetually dark burrows that subterranean animals inhabit, where air may also contain noxious gases like ammonia and methane in high concentrations^22–30^, comprise extreme habitats. Of the ~250 species that occupy or take shelter in burrows, only ~25 species, most of which are mole rats of the Family Talpidae, permanently live underground^31–36^. Because they are concealed and most of them pose a challenge of keeping and breeding them in the laboratory for study^9^, data on the biology of the fossorial- and the subterranean dwelling animals are largely lacking or incomplete. Only a few species have been comprehensively investigated^9,37^. Burda^9^ observed that the biology of the subterranean mammals has been well-investigated only in 10 out of the 54 species.

**Figure 1:**
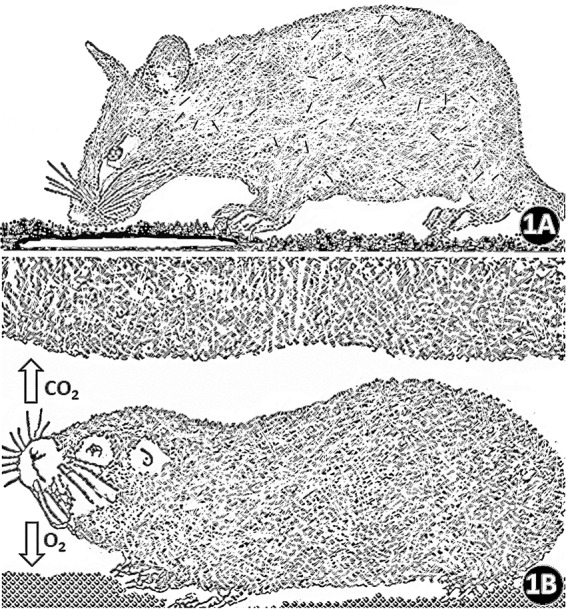
(**A**,**B)** Drawings of the semifossorial African giant pouched mole rat (*Cricetomys gambianus*) **(A)** which partly inhabits open burrows and the underground dwelling (subterranean) Nigerian mole rat (*Cryptomys foxi*) **(B)**. Compared to the air above the ground, that in the underground burrows generally contains relatively high concentrations of carbon dioxide (CO_2_) and low ones of oxygen (O_2_). (Figures drawn by the investigators).

The exacting environmental conditions in the underground burrows has compelled certain structural, functional, biochemical and genetic adaptations^1,4,9,28,29,31,38–43^. For example, the external morphologies of the burrow dwelling rodents include cylindrical (sausage-like) bodies, short and stout limbs, very small or abscent external ears, loss of eyes or poor vision and prehensile incisors for digging and biting off tubers (Fig. 1A,B): the adaptive convergence among the burrow dwelling animals epitomizes Darwin’s principle of evolution by natural selection^2,4,40,44^. It shows that underground dwelling animals are appropriate models for studying environmentally driven evolutionary and adaptive processes. Regarding aging and tumor resistance, the African naked mole rat, *Heterocephalus glaber*, is unique: it is the longest-living rodent with a maximum lifespan of 32 years^45–53^ and is particularly resistant to cancer^37,47,54–58^. Burda^9^ predicted that ‘subterranean mammals may become unique laboratory and model animals of the next generation’.

The structure of the lung of the semifossorial African giant pouched rat, *Cricetomys gambianus*, has hitherto been investigated only at gross and histological levels^59,60^ but that of underground dwelling Nigerian mole rat, *Cryptomys foxi*, has not. The only subterranean mole rats of which the lungs have been comprehensively morphometrically investigated are the African garden mole rat (*Tachyoryctes splendens*) and the naked mole rat (*H. glaber*)^61,62^ and the Middle Eastern blind mole rat (*Spalax Ehrenbergi*)^63^. Here, the morphology and the morphometry of the lungs of *C. gambianus* and *C. foxi* were investigated and compared to determine whether pulmonary specializations, which may permit subterranean life under low O_2_ concentrations and high ones of CO_2_, exist between the two species of rodents. The data were compared with those of other surface-, semifossorial- and subterranean dwelling rodents that have previously been investigated to similar extents.

From molecular through genetic to community levels, comparative biology highlights natural variation with the purpose of recognizing and understanding the structural and the functional basis of the designs of lifeforms and the pivotal survival role(s) that organisms play in ecosystems. Comparisons of two species or populations of animals that differ in certain attributes like body mass, genetic make ups, morphological and physiological properties, lifestyles pursued and habitats occupied have been performed for a long time^64–71^. Such studies have been used to explicate the consequences of the interactions between the phenotype and the environment, means and processes by which evolutionary adaptations are envisaged to occur^72–75^. Nearly three decades ago, from statistical- and evolutionary standpoints, Garland and Adolph^66^ argued that ‘limitations’ exist in comparisons between two species or populations of animals where interpretation(s) of adaptation(s) is/are made. Such studies have continued to be performed to characterise, differentiate and explain traits between species and populations, including supposed adaptive features^76–79^. Here, where we have cautiously termed the significantly higher pulmonary morphometric parameters of *C. foxi* (compared to the lower ones of *C. gambianus*) as ‘specializations’ instead of ‘adaptations’. If two species or populations comparisons in which relative differences are inferred to be adaptations are ultimately proven to be experimentally flawed, studies like ours will need to be re-evaluated.

## Results

### Morphology

**Figure 2:**
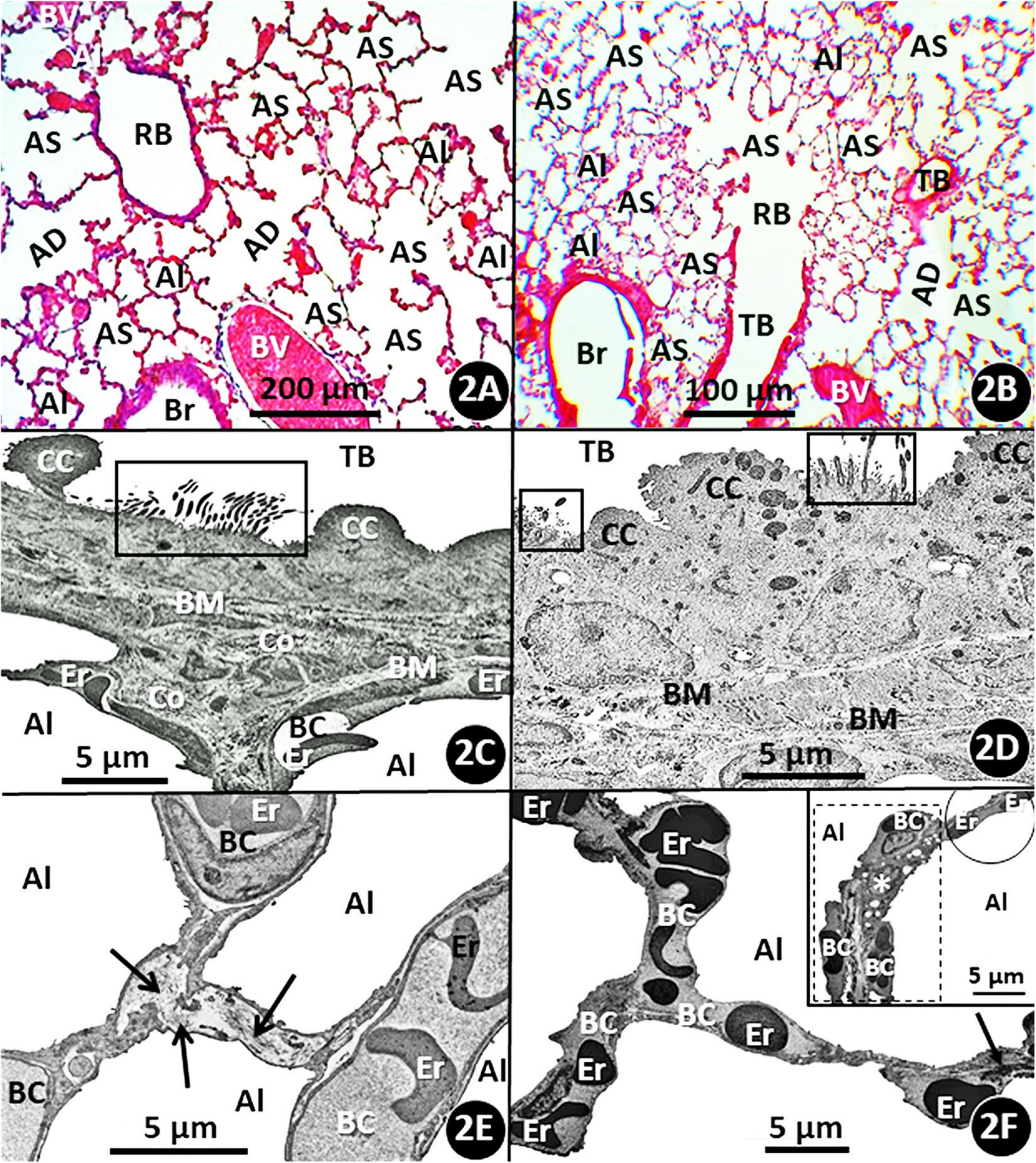
(**A–F**) Histological sections stained with hematoxylin and eosin showing the various structural components of the lungs of the semifossorial African giant pouched mole rat (*Cricetomys gambianus*) **(A)** and that of the underground-dwelling (subterranean) Nigerian mole rat (*Cryptomys foxi*) **(B)**. BV, blood vessels larger than blood capillaries; Br, bronchus; TB, terminal bronchus; RB, respiratory bronchiole; AD, alveolar duct; AS, alveolar sac; Al, alveolus. Transmission electron micrographs of the lungs of *C. gambianus* **(C)** and *C. foxi* **(D)** showing the epithelium that lines the terminal bronchus (TB). Boxed areas, ciliated epithelial cells; CC, Clara (serous) cells; BM, basement membrane; Co, collagen fibers; Er, erythrocytes; Al, alveolus; BC, blood capillary. Transmission electron micrographs of the lung of *C. gambianus* **(E)** and that of *C. foxi* **(F)** showing interalveolar septa that separate the alveoli (Al). The blood capillaries (BC) which contain erythrocytes (Er) are found in the interalveolar septa. Arrows, collagen fibres. The insert on Fig. 3F shows a double capillary system, where blood capillaries (BC) are exposed to air only on one side (dashed ‘boxed’ area) and a single capillary one where blood capillaries are exposed to air on both sides of the interalveolar septum (circle) of the parenchyma (gas exchange tissue) of the lung of *C. foxi*. Er, erythrocytes contained in the blood capillaries; Al, alveoli; asterisk, a type-II (granular) pneumocyte.

**Figure 3:**
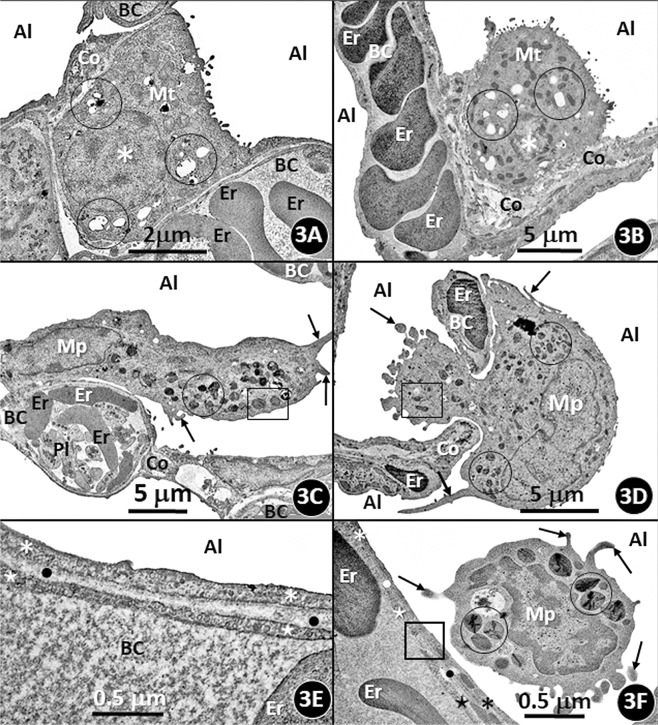
(**A–F**) Transmission electron micrograph of the lungs of the semifossorial African giant pouched mole rat *Cricetomys gambianus* **(A)** and that of the underground dwelling (subterranean) Nigerian mole rat *Cryptomys foxi* **(B)** showing type-II (granular) pneumocytes (asterisks). BC, blood capillaries containing erythrocytes (Er); circles, clear areas which in life form the osmiophilic lamellated bodies which are the precursors of the surfactant; Mt, mitochondria; Al alveoli; Co, collagen fibers. Transmission electron micrographs of the lungs of *C. gambianus* **(C)** and that of *C. foxi* **(D)** showing alveolar macrophages (Mp) located on the alveolar surface (**C**) and one passing through the interalveolar pore (**D**). Encircled areas, electron dense bodies that constitute the lysosomes; boxed areas, clusters of mitochondria; arrows, filopodia; BC, blood capillaries containing erythrocytes (Er); Pl, platelets; Co, collagen fibers; Al, alveoli. Transmission electron micrographs of the blood-gas barriers of the lungs of *C. gambianus* **(E)** and that of *C. foxi* **(F)** showing the blood-gas (tissue) barrier that comprises an epithelial cell (asterisk), a common basement membrane (dots) and an endothelial cell (stars). BC, blood capillary; Er, erythrocytes; Al, alveolus. On (**F**), an alveolar macrophage (Mp) lies next to the blood-gas barrier (boxed area). The arrows and the circles respectively show filopodia and lysosomes of the alveolar macrophage.

The morphological features of the lungs of the African giant pouched rat (*Cricetomys gambianus*) and those of the Nigerian mole rat (*Cryptomys foxi*) are comparatively shown respectively on Figs. 2A–F and 3A–F. In *C. gambianus*, the right lung comprises four lobes while the left one is undivided and in *C. fox* there are four lobes on the right lung and three of them on the left one. In the two rodent species, the lungs comprise two main structural parts, namely the parenchyma (exchange tissue) and the nonparenchyma: the former comprises respiratory bronchioles, alveolar ducts, alveolar sacs and alveoli while the later consists of the large blood vessels, i.e., those larger than blood capillaries, and large airways (bronchi) that bifurcate to terminate in terminal bronchioles (Fig. 2A,B). The main airways are lined by an epithelium that largely consists of ciliated cells amongst which goblet (mucous secreting) cells are scattered. In the terminal bronchioles of the lungs of the two species, Clara or serous cells are interspersed amongst the ciliated cells (Fig. 2C,D). The respiratory bronchioles are associated with several alveoli before they terminate in alveolar ducts and the alveolar sacs open into alveoli (Fig. 2A,B): the alveoli are rather hexagonal in shape and are separated by interalveolar septa. Collagen fibers are found in the interalveolar septa (Fig. 2E,F) and blood capillaries, which are located in the interalveolar septa and protrude into the alveolar (air) spaces. Inter-alveolar pores connect adjacent alveoli. In *C. foxi* (but not in *C. gambianus*), occasionally, the blood capillaries are exposed to air only on one side of the interalveolar septum (Fig. 2F - insert). The alveolar surface is covered by the thin (squamous) type-1 pneumocytes (Fig. 2E,F) and the rather cuboidal granular (type-II) pneumocytes which are dispersed over the respiratory surface (Fig. 3A,B). On our tissue preparations, the type-II cells contained clear spaces which in life form the osmiophillic lamellated bodies, the precursors of the surfactant. Alveolar macrophages (phagocytes) exist on the alveolar surface (Fig. 3C,D): the cells possess distinctive filopodia and numerous intracytoplasmic electron dense bodies, presumably lysosomes. The blood-gas (tissue) barrier consists of epithelial- and endothelial cells which are connected back-to-back across a common basement membrane (Fig. 3E,F).

### Morphometry

The morphometric data of the lungs of *C. gambianus* and *C. foxi* are shown on Tables 1–3, comparisons between the body mass normalized (weighted) values of the two species are shown on Table 4 and comparison of the species studied here with the other rodent species that have hitherto been studied on Table 5. The body mass, the lung volume, the volume densities and the absolute volumes of the main structural components of the lungs of *C. gambianus* and *C. foxi* are shown on Table 1: the volume densities of the parenchyma, the blood vessels larger than the blood capillaries and the airways varied between the species. The volume densities of the structural components of parenchyma of the lungs of *C. gambianus* and *C. foxi* are shown on Table 2: the volume densities of the alveoli, the blood capillaries, the red blood cells, the tissue of the blood-gas (tissue) barrier, the tissue of the interalveolar septum that is not involved in gas exchange and the volume of the blood in the pulmonary blood capillaries differed between the two rodent species. Table 3 shows the surface areas and the thicknesses of the different parts of the air-hemoglobin pathways in the lungs of *C. gambianus* and *C. foxi*: the alveolar surface area, the capillary endothelial surface area, the surface area of the blood-gas (tissue) barrier, the surface area of the erythrocytes, the harmonic mean thicknesses of the blood-gas (tissue) barrier, the thickness of the air-hemoglobin barrier and the arithmetic mean thickness of the blood-gas (tissue) barrier contrasted between the two species. The mass-specific, i.e., the body mass normalized (weighted) pulmonary morphometric parameters of *C. gambianus* and *C. foxi* are compared on Table 4: the volume of the lung of *C. foxi* exceeded that of *C. gambianus* by a factor of 2.7; the surface area of the blood-gas (tissue) barrier by a factor of 3.4; the pulmonary capillary blood (Vc) by a factor of 2; the harmonic mean thickness of the blood-gas tissue barrier (τht) was ~29% thinner; the diffusing capacity of the blood-gas (tissue) barrier for oxygen (Dto_2_) by a factor of 5; the membrane diffusing capacity (Dmo_2_) by a factor of 5.1 and; the total morphometric pulmonary diffusing capacity (DLo_2_) was 2.3 times greater.

**Table 1:** Body mass, lung volume, volume densities (%) and the absolute volumes [cm^3^] of the main structural components of the lungs of the African giant mole rat (*Cricetomys gambianus)* and the Nigerian mole rat (*Cryptomys foxi*).

Specimen number	Body mass (g)	Lung volume (cm^3^)	Parenchyma (%) [cm^3^]	Blood vessels larger than capillaries (%) [cm^3^]	Airways (%) [cm^3^]
*Cricetomys gambianus*					
1	680.12	5.03	(86.67) [4.36]	(6.08) [0.31]	(7.20) [0.36]
2	712.12	5.29	(86.19) [4.55]	(6.15) [0.33]	(7.66) [0,41]
3	678.30	4.81	(83.78) [4.01]	(8.19) [0.39]	(8.45) [0.41]
4	736.23	5.92	(86.40) [5.12]	(5.28) [0.31]	(8.32) [0.49]
5	697.74	5.48	(80.99) [4.44]	(8.58) [0.47]	(10.42) [0.57]
Mean ± S.D.	700.90 ± 21.57	5.31 ± 0.38	(84.73 ± 2.43 [4.49 ± 0.36]	(6.86 ± 1.44) [0.37 ± 0.06]	(8.41 ± 1.23) [0.45 ± 0.74]
*Cryptomys foxi*					
1	101.17	2.46 ± 0.06	(80.53) [1.98]	(12.53) [0.31]	(6.94) [0.17]
2	124.57	2.33 ± 0.02	(80.29) [1.87]	(11.76) [0.27]	(7.95) [0.19]
3	120.38	2.29 ± 0.03	(80.10) [1.83]	(11.21) [0.26]	(8.69) [0.20]
4	121.71	2.30 ± 0.02	(79.85) [1.84]	(12.07) [0.28]	(8.08) [0.18]
5	104.73	2.32 ± 0.03	(79.84) [1.85]	(11.80) [0.27]	(8.36) [0.20]
Mean ± S.D.	114.51 ± 10.74	2.34 ± 0.06	(80.12 ± 0.29) [1.87 ± 0.05]	(11.87 ± 0.48) [0.29 ± 0.01]	(8.01 ± 0.66) [0.18 ± 0.01]

**Table 2:** Volume densities (%) and absolute volumes [cm^3^] of the main structural components of the parenchyma (gas exchange tissue) of the lungs of the African giant mole rat (*Cricetomys gambianus)* and the Nigerian mole rat (*Cryptomys foxi*).

Specimen number	Alveoli	Blood capillaries	Red blood cells	Tissue of the blood-gas barrier	Tissue of the interalveolar septa not involved in gas exchange
*Cricetomys gambianus*					
1	(70.4%) [3.07]	(9.43%) [0.41]	(6.23%) [0.27]	(6.46%) [0.28]	(7.48%) [0.33]
2	(68.25%) [3.11]	(11.34%) [0.52]	(6.81%) [0.31]	(5.98%) [0.26]	(7.62%) [0.35]
3	(61.21%) [2.45]	(13.31%) [0.54]	(9.59%) [0.38]	(6.76%) [0.27]	(9.13%) [0.37]
4	(72.25%) [3.70]	(10.51%) [0.54]	(5.57%) [0.28]	(3.03%) [0.16]	(8.64%) [0.44]
5	(70.13%) [3.11]	(13.13%) [0.59]	(5.56%) [0.25]	(4.85%) [0.21]	(6.33%) [0.28]
Mean ± S.D.	(68.45 ± 4.82%) [3.09 ± 0.39]	(11.54 ± 1.34%) [0.25 ± 0.06]	(6.75 ± 0.49%) [0.29 ± 0.05]	(5.41 ± 1.32%) [0.24 ± 0.04]	(7.85 ± 1.18%) [0.35 ± 0.05]
*Cryptomys foxi*					
1	(77.87%) [1.54]	(7.26%) [0.14]	(4.83%) [0.10]	(5.19%)[0.10]	(4.85%) [0.10]
2	(71.95%) [1.35]	(11.57%) [0.22]	(4.97%) [0.09]	(5.49%)[0.10]	(6.02%) [0.11]
3	(75.31%) [1.38]	(9.73%) [0.18]	(5.14%) [0.09]	(4.40%)[0.08]	(5.42%) [0.10]
4	(75.11%) [1.38]	(9.45%) [0.17]	(4.98%) [0.09]	(6.47%) [0.12]	(3.99%) [0.08]
5	(77.14%) [1.43]	(10.62%) [0.20]	(4.45%) [0.08]	(5.04%) [0.09]	(2.75%) [0.05]
Mean ± S.D.	(75.56 ± 2.07%) [1.42 ± 0.07]	(9.56 ± 1.61%) [0.18 ± 0.03]	(4.91 ± 0.29%) [0.09 ± 0.01]	(5.13 ± 0.45%) [0.10 ± 0.01]	(4.84 ± 1.38%) [0.08 ± 0.02]

**Table 3:** Surface areas [cm^2^] of the components of the air-haemoglobin pathway of the lungs of the African giant pouched rat (*Cricetomys gambianus)* and the Nigerian mole rat (*Cryptomys foxi*); mass-specific (body mass normalized) values (cm^2^/g) and thicknesses of the blood-gas (tissue) barrier (τht), the total barrier, i.e., the thickness of the blood-gas barrier and plasma layer (τhtb) and the arithmetic mean thickness of the blood-gas (tissue) barrier (τt).

Specimen	Alveolar surface area	Capillary endothelial surface area	Blood-gas barrier surface area	Red blood cell surface area	τht (μm)	τhtb (μm)	τt (μm)
*Cricetomys gambianus*							
1	[5188.81] (7.63)	[3701.51] (5.44)	[3923.54] (5.77)	[3094.42] (4.54)	0.309	0.618	0.862
2	[6675.02] (9.37)	[4484.93] (6.30)	[4792.83] (6.73)	[4959.50] (6.96)	0.379	0.472	1.075
3	[5257.11] (7.75)	[3928.86] (5.49)	[4047.77] (5.97)	[4064.77] (5.97)	0.334	0.449	0.912
4	[7132.62] (9.69)	[3750.25] (5.09)	[43975.27] (5.40)	[44761.04] (6.47)	0.275	0.469	0.801
5	[6968.94] (9.99)	[4943.31] (7.08)	[5172.51] (7.41)	[5420.31] (7.77)	0.284	0.512	0.813
Mean ± SD	[6244.50 ± 847.15] (8.89 ± 0.99)	[4161.77 ± 479.72] (5.88 ± 0.72)	[4382.23 ± 506.16] (6.26 ± 0.72)	[4450.01 ± 810.35] (6.34 ± 1.08)	0.332 ± 0.04	0.504 ± 0.06	0.892 ± 0.09
*Cryptomys foxi*							
1	[3071.85] (30.36)	[2158.20] (21.33)	[2200.31] (21.75)	[1947.55] (19.25)	0.296	0.452	1.204
2	[3258.63] (26.16)	[2174.60] (17.46)	[2136.77] (17.15)	[1720.64] (13.81)	0.251	0.455	1.211
3	[3149.36] (27.88)	[22351.49] (19.53)	[2398.72] (19.92)	[1863.83] (15.48)	0.249	0.398	0.998
4	[2985.58] (24.53)	[2737.85] (22.49)	[2781.49] (22.85)	[2343.81] (19.26)	0.259	0.508	1.321
5	[3017.29] (28.81)	[2470.53] (23.59)	[2501.08] (23.88)	[1533.17] (14.64)	0.236	0.459	1.209
Mean ± SD	[3096.54 ± 98.25] (27.20 ± 2.09)	[2378.53 ± 213.75] (20.88 ± 2.17)	[2403.67 ± 230.15] (21.11 ± 2.38)	[1881.80 ± 270.41] (16.49 ± 2.32)	0.258 ± 0.02	0.454 ± 0.04	1.189 ± 0.12

Units: Surface areas, cm^2^; mass specific surface area, cm^2^/g.

**Table 4:** Comparisons of normalized (specific) pulmonary morphometric parameters (values) of the lungs of the African giant mole rat (*Cricetomys gambianus*) and the Nigerian mole rat (*Cryptomys foxi*).

Structural parameter	African giant rat *(C. gambianus)*	Nigerian mole rat *(C. foxi)*	Ratios of morphometric parameters of *C. foxi* divided by those of *C. gambianus*
VL/W (cm^3^/g)	0.0075	0.0204	2.720
St/W (cm^2^/g)	6.25	20.99	3.360
St/Vp (mm^2^.mm^3^)	97.59	128.50	1.317
Vc/St (cm^3^/m^2^	1.181	1.235	1.046
Vc/VL (cm^3^/cm^3^)	0.1520	0.115	0.757
Vc/W (cm^3^/kg)	1.157	2.357	2.037
τht (µm)	0.332	0.258	0.777
Dto_2_/W (mlo_2_/sec./mbar/kg}	0.0778	0.3877	4.983
Dmo_2_/W (mlo_2_/sec./mbar/kg)	0.0425	0.2160	5.082
Deo_2_/W (mlo_2_/sec./mbar/kg)	0.0448	0.0732	1.633
DLo_2_/W (mlo_2_/sec./mbar/kg)	0.0231	0.0533	2.307

Definitions of the abbreviations of structural parameters:

W, body mass; VL, volume of the lung; St surface area of blood-gas (tissue) barrier; Vp, volume of the parenchyma; Vc, volume of the pulmonary capillary blood; τht, harmonic mean of the blood-gas (tissue) barrier; Dto_2_, morphometric diffusing capacity of the blood-gas (tissue) barrier for oxygen; DMo_2_, membrane diffusing capacity or the morphometric diffusing capacity of the total barrier for oxygen; Deo_2_, morphometric diffusing capacity of the red blood cells for oxygen; DLo_2_, total morphometric pulmonary diffusing capacity of the lung for oxygen.

**Table 5:** Comparison of normalized (specific) pulmonary morphometric parameters (values) of the African giant rat (*Cricetomys gambianus*) and the Nigerian mole rat (*Cryptomys foxi*) investigated in this study with other underground- and surface-dwelling rodents.

Parameter/units	*C. gambianus*^#^	*C. foxi*^+^	*T. Splendens*^+^	*H. glaber*^+^	*S. ehrenbergi*^+^	*M. musculus*^•^	*R. rattus*^•^	*C. porcellus*^•^
VL/W (cm^3^/g)	0.0075	0.0230	0.041	0.039	0.0548	0.035	0.045	0.030
St/W (cm^2^/g)	6.25	20.99	31.08	23.3	4.6827	29.76	27.71	21.21
St/Vp (mm^2^/mm^3^)	97.59	128.50	86.62	77.01	—	—	—	—
Vc/St (cm^3^/m^2^)	1.181	1.235	0.878	1.056	1.4830	1.176	1.237	1.604
Vc/VL (cm^3^/cm^3^)	0.152	0.115	0.067	0.063	0.1201	0.101	0.076	0.110
Vc/W (cm^3^/kg)	1.157	2.357	2.728	2.463	6.5863	3.500	3.429	3.329
τht (μm)	0.332	0.258	0.203	0.243	0.363	0.290	—	—
τtb (μm)	0.504	0.454	—	0.251	0.6300	—	—	—
τt (µm)	0.890	1.189	0.678	1.091	1.040	—	1.228	—
DLo_2_ (mlO_2_/sec/mbar/kg)	0.0231	0.0533	0.0432	0.0389	0.0024	0.0507	0.0485	0.0417

Definitions of the abbreviations of the structural parameters:

W, body mass; VL, lung volume; St, surface area of the blood gas (tissue) barrier); Vp, volume of parenchyma; Vc, volume of pulmonary capillary blood; τht, harmonic mean of the blood-gas (tissue) barrier; τtb, harmonic mean of the total blood-gas barrier, i.e., the distance between the alveolar surface and the red blood cells; τt, arithmetic mean thickness; DLo_2_, total morphometric pulmonary diffusing capacity.

Mode of life:

^•^surface dwelling; ^#^fossorial lifestyle; ^+^subterranean (underground) dwelling.

Sources of data:

*C. gambianus and C. foxi (this study); T. splendens and H. glaber*^61^*; H. glaber*^62^*; M. musculus*^208^*; R. rattus*^209^*; C. porcellus*^210^*; S. ehrenbergi*^63^.

## Discussion

The etymology of the word ‘fossorial’ is the Latin one ‘fossor’ which means ‘digger’. It generally refers to an assortment of animals which have adapted to burrowing and living largely, but not exclusively, underground^2,31,44,80,81^. The term ‘subterranean’ designates animals that permanently live underground, i.e., in closed burrows, and rarely, if ever, come above ground. Here, we have deemed *C. gambianus* to be ‘semifossorial’ because it occupies open burrows part of the time and *C. foxi* ‘subterranean’ because it permanently dwells underground. The usage of these terms differs between investigators and authors. On the whole, subterranean animals inhabit hypoxic- and hypercapnic environments^28,36,39,82^. In burrows of the waterlogged soil occupied by *Spalax carmeli*, Shams *et al*.^29^ measured CO_2_ concentrations as high as 6.1% and those of O_2_ as low as 7.2%. The most extreme conditions in burrows so far reported are a minimum O_2_ concentration of 6% and a maximum CO_2_ concentration of 9%^83^^-p105^. Astonishingly, some animals living under such conditions can still perform the strenuous activity of digging^63,84–86^. The O_2_ tension which *S. ehrenbergi* tolerates is well-below that which exists at the top of Mount Everest and the CO_2_ tension is 200 times greater than that in the free atmosphere^23,29,87^. The naked mole rat, *H. glaber*, can tolerate five hour exposure to 80% CO_2_ concentration and 18 minutes of anoxia without suffering perceptible injury^88^. In surface dwelling mammals, extreme hypoxia and hypercapnia affects cardiac function, induces artificial hypothermia and torpor, reduces growth rate and lowers ventilatory rate^25,89–93^. Tomasco *et al*.^81^ designated the various adaptations that have developed in subterranean rodents as ‘adaptations of rodents to hypoxia and hypercapnia’. The traits have been well-characterized in the mole rats^4,9,29,36,61–63,87,94–97^.

The genus *Cricetomys* (to which *C. gambianus* belongs) is the most widely distributed rodent on tropical- and subtropical Africa^42,98,99^. It weighs as much as 2.8 kg^100,101^, grows to a body length of ~1 meter^102^ and is amongst the largest muroid species^100–102^. With a longevity of ~13.5 years^102,103^, it lives in colonies as large as 20 animals^101^. Because of its highly developed sense of smell^104^, *C. gambianus* has been effectively trained for mine detection^105,106^, some human communities use it for food^107^ and it is also kept as a pet^108^. The rodent has, however, earned the bad reputation of being a potential reservoir host for the zoonotic monkeypox virus^109,110^. *C. foxi*, is a subterranean rodent belonging to the family Bathyergidae and occupies tropical dry lowland grasslands, riverside woodlands and rocky places in colonies which excavate extensive burrows^111,112^. While gross anatomical and histological details on the structure of the respiratory system of *C. gambianus* exist^60,113^, *C. foxi* has not hitherto been investigated. Here, it was observed that while the lobulation of the lungs of the two species differed markedly, at histological- and ultrastructural levels, the organs were quite similar. They also resembled those of surface- and underground dwelling rodents^114,115^. The undivided left lung of *C. gambianus*, which was also reported in the same rodent by Ibe *et al*.^60^, and the existence of three lobes on the left lung of *C. foxi* are not unique morphological features: various permutations in the lobulations of the lung occur in the mammalian lungs^61,62,113,116–121^. Ostensibly, there is no evidence showing that lobulation of the lung is of functional consequence^122–134^. In their gross and histological study, Ibe *et al*.^59^ reported that the respiratory system of *C. gambianus* was ‘poorly’ developed and deduced that it was much less efficient in gas exchange compared to those of other small rodents. Although the interpretation is supported by this study, the conclusion reached (by Ibe *et al*.^59^) in the investigation was not merited by the simple data adduced. For both *C. gambianus* and *C. foxi*, the numerous pulmonary goblet cells and alveolar macrophages should constitute an important part of the pulmonary defense system^124,135,136^: the air in the burrows is presumably highly polluted and loaded with pathogens and particulates. Interestingly, a double blood capillary arrangement was observed in some parts of the interalveolar septa of the lung of *C. foxi* but not in those of *C. gambianus*. Similar design was reported in the lung of *H. glaber* by Maina *et al*.^61,62^ where the septal architecture was ascribed to possible neotenic or paedomorphic phylogenetic status of the species which is the most basal of the African mole rat lineages^46^. Among the air-breathing vertebrates, double capillary arrangement characterizes the amphibian^126,128,137,138^ and the reptilian^128,138,139^ lungs while in the mammalian lung the design normally occurs during the early stages of embryonic development^140^.

Significant differences were found between the pulmonary mass-specific morphometric parameters of *C. gambianus* and *C. foxi* (Table 4): the volume of the lung of *C. foxi* was 2.7 times larger, the respiratory surface area 3.4 times greater, the volume of the pulmonary capillary blood 2 times higher, the harmonic mean thickness of the blood-gas (tissue) barrier (τht) ~29% thinner, the pulmonary morphometric diffusing capacity of the blood-gas (tissue) barrier for oxygen (Dto_2_) 5 times greater, the membrane diffusing capacity for O_2_ (Dmo_2_) 5.1 times greater and the inclusive total pulmonary morphometric diffusing capacity for O_2_ (DLo_2_) 2.3 times higher. Comparison of the values of *C. gambianus* and *C. foxi* with those of other surface-, semifossorial- and subterranean dwelling rodents that have hitherto been investigated (shown on Table 5) indicates that different pulmonary morphometric specializations exist among the rodents. The lowest mass-specific lung volume occurs in *C. gambianus* (0.0075 cm^3^/g) and highest value in *S. ehrenbergi* (0.055 cm^3^/g); *S. ehrenbergi* has the lowest mass-specific surface area of the blood-gas (tissue) barrier (4.68 cm^2^/g) and the greatest one of 31.1 cm^2^/g occurs in *T. splendens*; the highest mass-specific pulmonary capillary blood volume is in *S. ehrenbergi* (6.59 cm^3^/kg^−1^) and the lowest one in *C. gambianus* (1.16 cm^3^/kg^−1^); the τht is smallest in *T. splendens* (0.203 µm) and greatest in *Carvia porcellus* (0.402 µm) and; the mass-specific DLo_2_ is highest in *C. foxi* (0.053 mlo_2_/sec/mbar/kg) and lowest in *S. ehrenbergi* (0.002 mlo_2_/sec/mbar/kg). Among the rodent species, regarding the τht, generally, the thinnest barriers occur in the subterranean mole-rats (Table 5). Pulmonary structural specializations, however, only form part of a broad suite of adaptations that have evolved for subterranean life^4,29,36,61–63,86,94–103,141,142^. Between the subterranean *S. ehrenbergi* and the above-ground dwelling rat, *Rattus norvegicus*, structural and functional differences correlate with the hypoxic stress and the greater vascularization of muscle tissue^29,143,144^. Genetic adaptation to life underground has been determined in *S. ehrenbergi*^145–147^. According to Skinner and Chimimba^112^ and Ewer^136^, at night, the omnivorous *C. gambianus* spends 67% of the time resting, 9.5% active in the burrows and 23.4% active outside: the most active times are between 1900 to 2200 hours and between 0100 to 0500 hours. Compared with those of *C. foxi*, the inferior pulmonary morphometric properties of the lung of *C. gambianus* (this study) is possibly attributable to its much larger body size and possibly lower metabolic rate, less energetically costly lifestyle where it spends most of the time resting^112^ and the fact that it retreats into less complex open burrows^136^ which are possibly ventilated with air from above the ground.

Burrow (subterranean) dwelling animals are not the only group of animals that cope with hypoxia in life. For animals living in and breathing water, O_2_ concentrations in aquatic environments have been declining for the past 50 years and are expected to continue falling over the 21^st^ century^148–152^. Animals that live and operate under the hypoxic environment conditions of the high altitude have acquired certain physiological adaptations that increase O_2_ acquisition^153–155^. For the human beings, acute exposure to hypobaric-hypoxia, equivalent to that at the top of Mt. Everest (altitude 8,848 m, barometric pressure 33.7 kPa), is injurious to most unacclimatized people without an auxiliary source of O_2_. However, some healthy people working at the top of their aerobic capacity have climbed to the top of some of the highest peaks of the Himalayas^156–158^. Interestingly, high altitude hypoxia does not appear to restrict the distribution of reptiles and amphibians^159^: 11 species of frogs live between an elevation of 3.7 and 5.2 km and the highest altitude dwelling reptiles, namely the Himalayan ground skinks, inhabit altitudes from 0.15 to 5.5 m^160^. Behavioral, physiological and morphological adaptations have allowed the frog, *Telmatobius coleus*, to occupy the high elevation Lake Titicaca on the border between Peru and Bolivia (altitude 3.8 km)^161–163^: its skin is well-vascularized and corrugated to increase the respiratory surface area and its blood has small, plentiful erythrocytes (the greatest number among amphibians), high hemoglobin concentration and high hematocrit. Among vertebrates, the capacity of birds to survive and also fly, i.e., exercise, at extreme altitude is exceptional. The cost of level flight in the rarefied air of the high altitude is additionally much greater than that at sea level^164^. Compared to mammals, birds have notably greater tolerance of hypoxia^165–167^. Many species have been tracked migrating at an altitude of over 6 km^168^ and the outstanding high altitude flyers include the bar-headed goose (*Anser indicus*)^169–176^, the Andean goose (*Chloephaga melanoptera*)^169,177^, the Tibetan chicken (*Gallus gallus*)^178^ and the Ruppell’s griffon vulture (*Gyps rueppellii*)^179^. A griffon vulture collided with a commercial jet craft at an altitude of 11.3 km (37,000 ft) over Abidjan (Cote d’Ivoire, West Africa)^180^. The bar-headed geese migrate from the Indian subcontinent and cross the highest of the Himalayan Peaks to winter out and breed around the great lakes of the south-central Asia, an average elevation of 5.5 km^167,173–176,181^: the birds take off from near sea level and without acclimatizing almost directly reach an altitude of ~10 km^169,181–183^. At the elevation they cross the peaks, the barometric pressure is ~31 kPa and the PO_2_ in the dry air is 6.5 kPa^184^. The bar-headed goose can tolerate extreme hypoxia where the O_2_ tension in the inspired air is ~2.8 kPa, a value equivalent to that at an elevation of 12 km^169^. Assuming that such birds maintain a constant body temperature (41 °C) and the inhaled air is warmed to that of the body and fully saturated with moisture (water), the PO_2_ in the air reaching the respiratory surface should not exceed 4.9 kPa. The Andean goose, a large bird that lives at altitudes between 3 km and 6 km of the Andes^177^ has morphologically remarkably specialized lungs^185^: compared to most other birds the Andean geese have larger lung volumes, the respiratory surface area is greater, the blood-gas barrier is thinner and total pulmonary morphometric diffusing capacity is greater. An important respiratory physiological difference between birds and mammals is that to a relatively greater extent, birds tolerate hypocapnia^165,186^, an attribute that makes it possible for birds to hyperventilate (and that way increase O_2_ uptake) without suffering respiratory complications like blood pH imbalance, i.e., respiratory alkalosis. Birds tolerate high level of arterial hypoxemia fundamentally because their brain blood flow is not affected and in some cases even increases when the arterial blood PO_2_ drops to below 10 kPa^165,186–190^. In human beings, reduction of the arterial PCO_2_ to 1.3 kPa causes hypocapnic cerebral vasoconstriction where blood flow to the brain may decrease by as much as ~50%^191^. In the bar-headed goose, cerebral blood flow is not affected by decline in the arterial PCO_2_ to 0.9 kPa^186^. Animals that tolerate hypoxic conditions have developed various adaptations that increase O_2_ extraction from the environment they occupy and its distribution to the tissues and cells^153,178,192–196^.

In conclusion, this study shows that while the gross anatomical features of the lungs of *C. gambianus* and *C. foxi* differ, histologically and ultrastructurally they were rather similar and generally resemble those of other rodents. Various pulmonary morphometric specializations occur between the subterranean-, the fossorial- and the surface dwelling rodents. In the burrow (underground) dwelling ones, pulmonary structural refinements should enhance O_2_ acquisition under hypoxic and hypercapnic conditions.

## Materials and methods

The specimens of mole rats which were used in this study were collected in Nigeria in compliance with NIH Guideline for Care and Use of Laboratory Animals, 8^th^ edition (Revised 2010). The study was approved by the University of Nigeria (Nsukka) Animal Ethics Committee - approval reference number: UNN/FVM/VANAT/RE/17/F0013. Processing of tissues and analysis were conducted at the University of Johannesburg, South Africa.

### Collection of specimens

Five adult specimens of the semifossorial African giant pouched mole rat (*Cricetomys gambianus*) (which is also called the Gambian pouched rat) were captured at night in metal-cage traps between the months of January and March in their natural habitat. The traps were placed near the openings of the burrows which were located in farms and gardens within Nsukka Metropolis of South-Eastern Nigeria. Nsukka is a sub-urban area where the temperatures range from 21.17 to 32 °C, the rainy (wet) season lasts from March to October while the dry season runs from November to February and the relative humidity is ~70% during the rainy season and 20% during the dry season^197^. The soils in Nsukka area are sandy and clay^198,199^. They are prone to erosion and leaching from the heavy rains that fall in the region^199^.

Five adult specimens of both sexes of the subterranean Nigerian mole rat (*Cryptomys foxi*) were caught during the day between the months of February and March at Panyam, a rural farming area in the Mangu Local Government Area near Jos in the Plateau State of North Central Nigeria. The average altitude is 1,280 m above sea level^199^. The mole rats excavate extensive underground burrows which are dug through reddish basaltic soils that are derived from biotite-granites, alluvium, unconsolidated quaternary deposits and granite-gneiss^199^. The climate at Panyam is semi-temperate, with the average annual mean temperature of 22.6 °C: March is the hottest month. The rainy season occurs between April and October while the dry one runs from Mid-October to early April: the average annual rainfall is 1,232 mm (www.en.climate-data.org, 2017).

### Fixation of the lungs

The animals were euthanized by intraperitoneal injection of euthanase^®^ (thiopental sodium) into the brachial vein at a dosage of 20 mg/kg body mass and weighed. Next, the diaphragm was punctured on both sides of the thoracic cavity causing pneumothorax and subsequent collapse of the lung. An incision was made on the anterior aspect of the neck and the trachea exteriolized and cannulated. The lungs were fixed by intratracheal instillation with Karnovsky’s fixative which comprised 2.5% glutaraldehyde and 2% paraformaldehyde in 0.1 M phosphate buffer (pH 7.4 and osmolarity 395 mosmol/L) at a constant pressure head of 25 cm water with the animal in a supine position. When the fixative stopped flowing, a ligature was placed ahead of the cannula and the animals kept in a cold room for ~4 hours. Thereafter, the lungs were removed from the thoracic cavity and their volume determined by the weight displacement method^200^.

### Sampling of the lungs

The lungs were sampled according to the number of existing lobes. For *C. gambianus* there were four lobes on the right lung and the left one was not lobulated while *C. foxi* had four lobes on the right lung while three lobes existed on the left lung. Each lobe was cut transversely into four slices of equal thickness of ~1 cm. Two slices were picked randomly and one used for light microscopy (LM) and the other for scanning electron microscopy (SEM). The remaining two pieces were diced and eight pieces picked by random computer number generation for transmission electron microscopy (TEM). The inclusive sampling technique adopted here ascertained that all the parts of the lung were represented in the tissue preparations that were analyzed.

In stereological/morphometric studies, the main purpose of sampling is to acquire representative or unbiased tissue samples that on analysis provide data that reproduce the ‘in-life’ state. While cutting up the entire lungs and taking samples from them would be an easier more direct method, it would not have quite guaranteed that the tissue samples were representative. This was ascertained by taking samples from each lobe.

### Processing for tissues for LM, TEM and SEM

For LM, pieces of the lung were processed by standard tissue processing techniques^201^. Tissues were embedded in paraffin wax, the blocks cut at 7 μm thickness and the sections stained with hematoxylin and eosin. For each block, the first technically adequate section was used for LM study and morphometric analysis at that level. For TEM, the pieces of lung tissue were processed by conventional laboratory techniques^201^ and ultra-thin sections cut and stained with uranyl acetate and lead citrate for viewing on a Philips CM 10 TEM microscope at an accelerating voltage of 80 kV. For SEM, samples of the lung tissue were dehydrated with ethanol and exposed to two changes of hexamethyldisilazine (HMDS) (Sigma-Aldrich, St. Louis, MO, U.S.A). The samples were sputtered with gold-palladium complex and viewed on a TESCAN^®^ VEGA3 scanning electron microscope (Brno, Czech Republic) at an accelerating voltage of 8 kV.

### Morphometric analysis

In all applicable cases, the morphometric values were adjusted for the magnification at which the analysis and the measurements were made.

#### Determination of the volume densities and the absolute volumes of the main components of the lung

The volume density of the parenchyma and those of the components of the non-parenchyma, i.e., the blood vessels larger than blood capillaries and the main airways (bronchi), were determined by point-counting^128,129,202,203^ at a final magnification of ×100 using a 100-point Zeiss integrating graticule. The sufficiency of the number of sections analysed was ascertained from cumulative (summation) average graph plots and the adequacy of number of points counted was confirmed from a normogram given in Weibel^202^^-p.114^. For the volume densities, the number of points counted reached a relative accuracy of a standard error of below 5%. The absolute volumes of the structural components were calculated as the product of the volume density and that of reference space, which was the volume of the lung. In this study, a shrinkage constant of 39.1%, which was reported on the mammalian lung by Schneider and Ochs196^204^^-p. L334^, and the procedure of correcting for shrinkage outlined by Hsia *et al*.^205^^-p. 402^, were applied to adjust for the reduction of the volume of the lung tissue during processing.

#### Determination of the volume densities and the absolute volumes of the components of the parenchyma

For *C. gambianus*, where a total of 5 lobes comprised the respiratory organ, for each lobe, 1 block was picked at random from 10 blocks which were prepared for each lobe and sectioned. For each section, 6 electron micrographs were taken at a primary magnification of ×2,400 from a predetermined top left corner of the grid squares to avoid bias. For each specimen, a total of 30 micrographs was taken. The micrographs were enlarged to a final magnification of ×6,800 at which morphometric analyses were done. For *C. foxi*, where the respiratory organ comprised a total of 7 lobes, 42 electron micrographs were taken from the seven blocks which were randomly picked. The volume densities of the components of the parenchyma, i.e., the alveoli, the blood capillaries, the blood-gas (tissue) barrier and that of the parts of the interalveolar septum not involved in gas exchange were determined by point-counting using a quadratic lattice grid with 100 points^130,206^. The absolute volumes of the structural components were calculated from their volume densities and the volume of the parenchyma, i.e., the reference space.

#### Determination of surface densities and surface areas

The surface densities of the alveolar surface, the blood-gas tissue barrier, the capillary endothelium and the red blood cells were determined by intersection counting^130,202,206^. For the various structural components, the adequacy of the test line lengths was verified from a normogram given in Weibel^206^^-p.119,^. The surface areas were calculated as the product of the surface density and the volume of the parenchyma, the reference space.

#### Determination of the harmonic mean thicknesses of the blood-gas (tissue) barrier (τht) and the total barrier (τhtb)

The harmonic mean thicknesses were determined by intercept length measurement using a logarithmic scale^130,203,207^. Respectively, the sum of the reciprocals of the intercept lengths were adjusted by factors of 2/3 and 3/4 to correct for the obliqueness of sectioning and the curvature of the surface of the erythrocytes^203,207^.

#### Determination of the arithmetic mean thickness of the blood-gas (tissue) barrier (τt)

The τt of the blood-gas (tissue) barrier was calculated as the mathematical average of the sum of the lengths of the intercepts divided by the number of intercepts measured.

#### Morphometric diffusing capacities of structural components of the lung

The method of calculating the morphometric diffusing capacities of the various parts of the air-hemoglobin pathway, namely the blood-gas (tissue) barrier, the membrane and the total diffusing capacity, are outlined in Maina^130^, Weibel^206^ and Weibel *et al*.^207^. The Krogh permeation constant (Kto_2_) of 4.1 × 10^−10^ cm^2^/sec/mbar and the O_2_-uptake coefficient of the whole blood (ΘO_2_) of 1.87 × 10^−2^ mlO_2_/ml/sec/mbar were applied in the calculation of the relevant anatomical (morphometric) diffusing capacities.

## Data Availability

Data included in the manuscript.
